# TV Viewing and Physical Activity Are Independently Associated with Metabolic Risk in Children: The European Youth Heart Study

**DOI:** 10.1371/journal.pmed.0030488

**Published:** 2006-12-12

**Authors:** Ulf Ekelund, Søren Brage, Karsten Froberg, Maarike Harro, Sigmund A Anderssen, Luis B Sardinha, Chris Riddoch, Lars Bo Andersen

**Affiliations:** 1 Epidemiology Unit, Medical Research Council, Cambridge, United Kingdom; 2 Institute of Sport Science and Clinical Biomechanics, University of Southern Denmark, Odense, Denmark; 3 National Institute for Health Development, Estonian Centre of Behavioural and Health Sciences, Tartuu, Estonia; 4 Department of Sports Medicine, Norwegian University of Sport Sciences, Oslo, Norway; 5 Faculty of Human Movement, Technical University of Lisbon, Lisbon, Portugal; 6 London Sport Institute, Middlesex University, London, United Kingdom; London School of Hygiene and Tropical Medicine, United Kingdom

## Abstract

**Background:**

TV viewing has been linked to metabolic-risk factors in youth. However, it is unclear whether this association is independent of physical activity (PA) and obesity.

**Methods and Findings:**

We did a population-based, cross-sectional study in 9- to 10-y-old and 15- to 16-y-old boys and girls from three regions in Europe (*n* = 1,921). We examined the independent associations between TV viewing, PA measured by accelerometry, and metabolic-risk factors (body fatness, blood pressure, fasting triglycerides, inverted high-density lipoprotein (HDL) cholesterol, glucose, and insulin levels). Clustered metabolic risk was expressed as a continuously distributed score calculated as the average of the standardized values of the six subcomponents. There was a positive association between TV viewing and adiposity (*p* = 0.021). However, after adjustment for PA, gender, age group, study location, sexual maturity, smoking status, birth weight, and parental socio-economic status, the association of TV viewing with clustered metabolic risk was no longer significant (*p* = 0.053). PA was independently and inversely associated with systolic and diastolic blood pressure, fasting glucose, insulin (all *p* < 0.01), and triglycerides (*p* = 0.02). PA was also significantly and inversely associated with the clustered risk score (*p* < 0.0001), independently of obesity and other confounding factors.

**Conclusions:**

TV viewing and PA may be separate entities and differently associated with adiposity and metabolic risk. The association between TV viewing and clustered metabolic risk is mediated by adiposity, whereas PA is associated with individual and clustered metabolic-risk indicators independently of obesity. Thus, preventive action against metabolic risk in children may need to target TV viewing and PA separately.

## Abstract 2

A study of over 1,900 European children showed that TV viewing and physical activity in children are separately associated with obesity and metabolic risk.

## Editors' Summary

**Background.:**

Childhood obesity is a rapidly growing problem. Twenty-five years ago, overweight children were rare. Now, 155 million of the world's children are overweight, and 30–45 million are obese. Both conditions are diagnosed by comparing a child's body mass index (BMI; weight divided by height squared) with the average BMI for their age and sex. Being overweight during childhood is worrying because it is one of the so-called metabolic-risk factors that increase the chances of developing diabetes, heart problems, or strokes later in life. Other metabolic-risk factors are fatness around the belly, blood-fat disorders, high blood pressure, and problems with how the body uses insulin and blood sugar. Until recently, like obesity, these other metabolic-risk factors were seen only in adults, but now they are becoming increasingly common in children. In the US, 1 in 20 adolescents has metabolic syndrome—three or more of these risk factors. Environmental and behavioural changes have probably contributed to the increase in metabolic syndrome in children. As a group, they tend to be less physically active nowadays and they eat bigger portions of energy-dense foods more often. Increased TV viewing during childhood (and the use of other media such as computer games) has also been linked to increased obesity and to poorer health as an adult.

**Why Was This Study Done?:**

One popular theory is that TV viewing may affect obesity and other metabolic-risk factors by displacing PA. Instead of playing in the yard after school, the theory suggests, children laze about in front of the TV. However, there is limited evidence to support this idea, and health professionals need to know whether TV viewing and PA are related, and how they affect metabolic-risk factors, in order to improve children's health. In this study, the researchers examined the associations between TV viewing, PA, and metabolic-risk factors in European children.

**What Did the Researchers Do and Find?:**

The researchers enrolled nearly 2,000 children in two age groups from three areas in Europe. They measured the children's height and weight, estimated how fat they were by measuring skin fold thickness, measured their blood pressure, and examined the levels of glucose, insulin, and different fats in their blood. The children completed a computer questionnaire about the lengths of time for which they watched TV and how often they ate while doing so, and their PA was measured using a device called an accelerometer that each child wore for four days. When these data were analyzed statistically, the researchers found that TV viewing was slightly associated with clustered metabolic risk (the average of the individual metabolic-risk factors). This association was due to an association between TV viewing and obesity—the children who watched most TV tended to be the fattest children. However, TV viewing was not related to PA. The most active children were not necessarily those who watched least TV. Most importantly, PA was related to all individual risk factors except for obesity and with clustered metabolic risk. These associations were independent of obesity.

**What Do These Findings Mean?:**

These results suggest that TV viewing does not damage children's health by displacing PA as popularly believed. The finding that the association between TV viewing and clustered metabolic-risk factors is mediated by obesity suggests that targeting behaviours like eating while watching TV might be a good way to improve children's health. Indeed, the researchers provide some evidence that eating while watching TV is associated with being overweight, but the results of this post hoc analysis—one that was not planned in advance—need to be confirmed. Another limitation of the study is the possibility that the children inaccurately reported their TV watching habits. Also, because measurements of metabolic-risk factors were made only once, it is impossible to say whether TV viewing or lack of PA actually causes an increase in metabolic-risk factors.

Nevertheless, these results strongly suggest that promoting PA is beneficial in relation to metabolic-risk factors, but less so in relation to obesity in childhood. TV viewing and PA should be treated as separate targets in programs designed to reverse the obesity and metabolic-syndrome epidemic in children.

**Additional Information.:**

Please access these Web sites via the online version of this summary at http://dx.doi.org/doi:10.1371/journal.pmed.0030488.

- US Centers for Disease Control and Prevention, information on overweight and obesity
- International Obesity Taskforce, information on obesity and its prevention, particularly in childhood
- Global Prevention Alliance, details of international efforts to halt the obesity epidemic and its associated chronic diseases
- American Heart Association, information for patients and professionals on metabolic syndrome and children's health

## Introduction

Metabolic and cardiovascular disease (CVD) risk factors comprise a number of factors including disturbed insulin and glucose metabolism, hypertension, general and abdominal obesity, and dyslipidemia (elevated triglycerides and decreased inverted high-density lipoprotein [HDL] cholesterol levels). Until recently, these risk factors had been observed only in adulthood, but they have now also been reported in children [1–3]. Environmental and behavioural changes during the past decades, such as increased TV viewing and reduced physical activity (PA) levels, may have contributed to this phenomenon. Excessive TV viewing has been linked to obesity [4–8], although the effect size appears to be small [4]. Furthermore, an independent association between childhood TV viewing and adult health status at age 26 y has been suggested [9]. It is also possible that TV viewing may affect obesity and other metabolic-risk factors by displacing PA [10,11]; however, the empirical evidence for this conclusion is limited.

In order to improve understanding of these complex relationships and to assist with the development of preventive action, we aimed to examine the cross-sectional associations between TV viewing and objectively measured PA, with metabolic-risk factors, after adjusting for adiposity and other possible confounders, in a large population-based sample of 9- to 10-y-old and 15- to 16-y-old children from three distinct regions in Europe.

## Methods

### Study Design

The European Youth Heart Study (EYHS) is designed to examine the nature, strength, and interactions between personal, environmental, and lifestyle influences on CVD risk factors in a large population-based sample of children from diverse areas in Europe. The rationale, aims, study design, selection criteria, and sample size are described in detail elsewhere [12].

Briefly, at each study location, a defined population of children was identified, and from this population a two-stage cluster sample of children was randomly selected. The primary sampling unit was schools, and the secondary unit was classes within schools. A minimum of 20 schools were randomly selected from local authority lists within appropriate age, gender, and socio-economic strata using probability proportional to school size. The overall response rate was 73% of those that were eligible, and was similar across age and gender groups. Written informed consent was obtained from a parent or guardian, and the study procedures were explained verbally to all the children. Ethical approval for the study was obtained from the local research ethics committee in each study region.

The present cross-sectional study is from the baseline data collection performed between 1997 and 2000 and includes 1,092 9- to 10-y-old children (544 boys and 548 girls) and 829 15- to 16-y-old adolescents (367 boys and 462 girls) from three geographically defined areas in Europe (the city of Odense, Denmark, the city and surrounding rural areas of Tartu, Estonia, and the island of Madeira, Portugal). Thus, the children represented diverse cultural, socio-economic, and geographical environments. Children from the Oslo area, Norway, are also part of the EYHS; however, since fasting venous blood samples were not available from this study location, they are not included in the present report.

Data on anthropometric variables, sexual maturity, and biochemical indicators were available in all children (*n* = 1,921). Of these children, 1,702 (89%) had valid data on objectively measured PA and self-reported TV viewing. Complete data, including birth weight, smoking status, and parental socio-economic status (SES, highest education and income), were available in 1,485 children.

### Measurements

Weight and height were measured while the participants were wearing light clothing, without shoes, using standard techniques. Body-mass index (BMI) was calculated as weight (kg)/height^2^ (m^2^). Children were classified as overweight and obese according to age and gender-specific cut-off points [13]. Four skin-fold measurements (triceps, biceps, subscapula, and suprailiac) were taken on the left side of the body in duplicate or triplicate, according to the criteria described by Lohman et al. [14]. The two closest measurements were averaged, and the sum of the four skin folds was used as an indicator of adiposity.

Resting systolic and diastolic blood pressures were measured in the sitting position, after 5 min of sitting rest, with a Dinamap vital-signs monitor (GE Healthcare, http://www.gehealthcare.com). Five measurements were taken at 2-min intervals, and the means of the last three measurements were averaged and used for analysis.

Sexual maturity was assessed by the data collectors, using the five-stage scale for breast development in girls and pubic hair in boys, according to Tanner [15]. Time (h d^−1^) spent viewing TV, frequency of eating while viewing TV (five-point scale), and smoking status (yes or no) was obtained by self-reporting using a computer-based questionnaire [12]. The children answered the questions individually without any interaction with other children. The computers were placed in a quiet area of the testing room(s), and one researcher was available at all times in the case the children had any questions or should uncertainties arise. The researcher was instructed not to interfere with the children during the completion of the questionnaires, and the children had to answer each question before the next question appeared on the screen—which reduced the amount of missing data. Two questions were asked about the amount of time watching TV as follows: “How many hours of TV do you usually watch before school?”, and “How many hours of TV do you usually watch after school?”, and the data were thereafter summarized into one variable (h d^−1^). Children's birth weight and parental SES were obtained by self-reporting from the parents.

### Assessment of PA

Free-living PA was assessed with the ActiGraph activity monitor (formerly known as the CSA activity monitor model WAM 7164) (http://www.theactigraph.com) over two weekdays and two weekend days, as previously described [16]. Briefly, the children wore the accelerometer in an elastic waistband on the right hip during the daytime, except whilst bathing and during other aquatic activities. Activity data were stored on a minute-by-minute basis and were downloaded to a computer before analysis. A Microsoft Excel–based macro was used for data reduction and further analyses. The outcome variable was daily activity counts per minute (cpm), which is an indicator of the total volume of PA (i.e., average intensity of PA). This variable was derived by dividing total counts by monitoring time per day, and was averaged over the measurement period. We have previously shown that this variable is significantly correlated with PA energy expenditure obtained by the doubly-labelled water method [17]. Children who did not manage to record at least 600 min d^−1^ of activity for at least 3 d were excluded from further analyses.

### Biochemistry

Overnight-fasting blood samples were taken in the morning from the antecubital vein. Samples were aliquoted and separated within 30 min, before being stored at −80 °C until they could be transported to World Health Organization (WHO)–certified laboratories for analysis*.* Samples from Denmark and Estonia were measured in one laboratory (Bristol, United Kingdom), whereas samples from Portugal were measured separately in a second laboratory (Cambridge, United Kingdom). HDL cholesterol, and triglycerides were measured by enzymatic methods in all samples (Olympus Diagnostica, http://www.olympus-diagnostica.com). Glucose was analysed using the Hexokinase method, measured on an Olympus AU600 auto-analyser in all samples (Olympus Diagnostica). In the Danish and Estonian samples, insulin was analysed using enzyme immunoassay (micro-titer plate format, Dako Diagnostics, http://www.dako.co.uk). In the Portuguese samples, plasma-specific insulin was determined by two-site immunometric assays with either ^125^I or alkaline phosphatase labels. Cross-reactivity was <0.2% with intact proinsulin at 400 pmol l^−1^ and <1% with 32–33 split proinsulin at 400 pmol l^−1^. Inter-assay coefficient of variance was 6.6% at 28.6 pmol l^−1^ (*n* = 99), 4.8% at 153.1 pmol l^−1^ (*n* = 102), and 6.0% at 436.7 pmol l^−1^ (*n* = 99), respectively. Between-laboratory correlations for 30 randomly selected samples analysed at both laboratories were 0.94–0.98. However, before the analyses could be carried out, all biochemical data were standardized to the mean (*z*-score) by study location, gender, and age group.

### The Metabolic-Risk Score

Broadly based on the definition proposed by WHO [18], we constructed a standardized continuously distributed variable (standardized metabolic-risk score, zMS) for clustered metabolic risk, which we have described in detail previously [19–21]. This variable was derived by standardizing and then summing the following continuously distributed metabolic-syndrome components: the sum of four skin folds, hypertension (average of systolic blood pressure and diastolic blood pressure), hyperglycaemia (fasting plasma glucose), insulin resistance (fasting insulin), inverted fasting HDL cholesterol, and hypertriglyceridemia, to create a *z*-score. We also calculated a clustered metabolic-risk score without the adiposity component (i.e., the sum of four skin folds) to examine whether the associations between the main exposures (TV viewing and PA) and clustered metabolic risk were mediated by adiposity. This score is referred to as zMS-Ob (standardized metabolic-risk score excluding the adiposity component). The purpose of using a continuously distributed variable was to maximize statistical power [22].

### Statistical Procedures

Fasting insulin and the sum of four skin folds were logarithmically transformed (ln) owing to their skewed distributions (geometric mean and reference intervals [1.96 × standard deviation] are presented in the results). Differences between genders, study locations, and age groups were tested by analysis of variance (ANOVA). Relationships between variables were assessed with correlation and partial correlation coefficients. The independent associations between TV viewing, and PA with the individual metabolic-risk factors were tested by generalized linear models, adjusting for gender, age group, study location, birth weight, sexual maturity, smoking status, and parental SES. We first assessed whether TV viewing, PA, and adiposity were associated with each individual phenotype per se in separate models. We thereafter assessed the independent associations between TV viewing and PA with each individual phenotype after further adjustment for adiposity (when adiposity was not the outcome of interest). Model building was performed by first introducing TV viewing, then PA, and thereafter testing for the interaction between these variables. We then tested whether TV viewing and PA were independently associated with the clustered metabolic-risk score in two models. The first model (adiposity-dependent) included all individual risk factors and was adjusted for the confounders described above.

In the second model (adiposity-independent), the adiposity component was excluded from the outcome, and adjustments were made for the sum of four skin folds (as a confounder) in addition to all other confounders. To examine whether gender and age group modified the associations between exposure variables (TV viewing and PA) and outcomes, interaction terms (gender × TV viewing, gender × PA, age group × TV viewing, and age group × PA) were included. All data were analysed in their continuous form, although data are stratified by quartiles of TV viewing and PA for illustrative purposes. Statistics were analysed with SPSS for Windows (Version 11.0) and a *p*-value < 0.05 denoted statistical significance. Values reported are means ± standard deviation unless otherwise stated.

## Results

The characteristics of the participants are shown in Table 1. Overall, 85% of the children were categorized as normal weight, 12% were overweight, and 3% were obese.

**Table 1:**
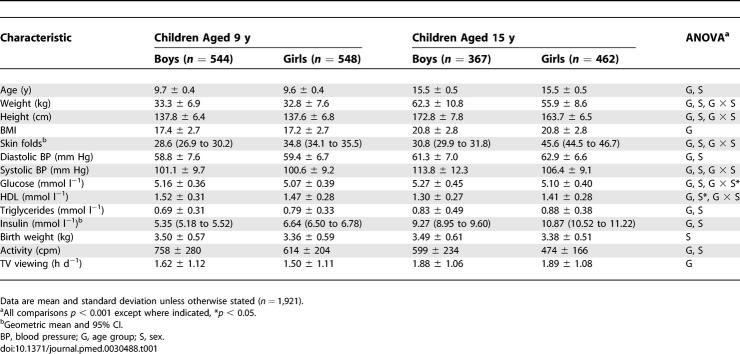
Descriptive Characteristics of Participants

**Figure 1:**
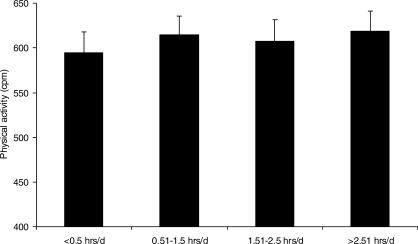
Objectively Measured PA (Mean 95% Confidence Interval [CI]) Stratified by Quartiles of TV Viewing (*n* = 1,702) Data are adjusted for gender, study location, age group, sexual maturity, smoking status, and parental SES.

TV viewing was not correlated with PA (*r* = 0.013, *p* = 0.58), and this nonsignificant correlation persisted after further adjustment for gender, age group, and study location (partial *r* = 0.01, *p* = 0.66), indicating that PA and TV viewing are separate entities. Figure 1 shows the total volume of PA stratified by quartiles of TV viewing adjusted for gender, study location, age group, sexual maturity, smoking status, and parental SES.

In Table 2, the separate associations between TV viewing, PA, and adiposity with individual risk factors are shown. All outcomes in Table 2 are expressed as standard deviation scores. TV viewing was significantly and positively associated with adiposity (*p* = 0.021) and fasting insulin (*p* = 0.013), after adjusting for gender, age group, study location, sexual maturity, smoking status, birth weight, and SES. In contrast, PA was not associated with adiposity (*p* = 0.18), but was significantly and independently associated with fasting insulin, fasting glucose, systolic and diastolic blood pressure (all *p* < 0.0001), and fasting triglycerides (*p* = 0.009), after adjusting for the same confounders as above. Adiposity was significantly and positively associated with fasting insulin (*p* < 0.0001), triglycerides (*p* < 0.0001), systolic blood pressure (*p* < 0.0001), and diastolic blood pressure (*p* = 0.002), after adjustment for the same confounders as above.

**Table 2:**
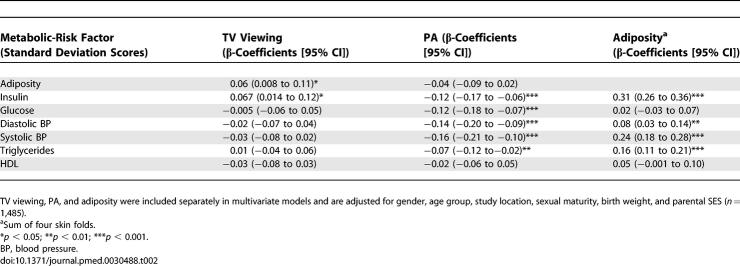
Associations of TV Viewing, Objectively Measured PA, and Adiposity with Individual Metabolic-Risk Factors

**Figure 2:**
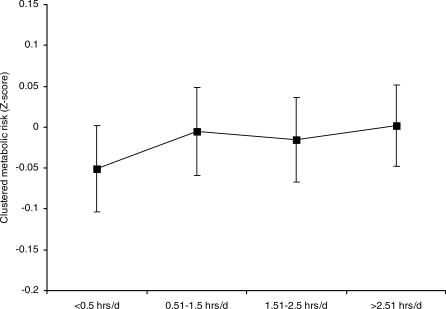
The Clustered Metabolic-Risk Score (Mean 95% CI) Stratified by Quartiles of TV Viewing (*n* = 1,485) Data on TV viewing are adjusted for PA, gender, study location, age group, sexual maturity, smoking status, birth weight, and parental SES (*p* = 0.053 in continuous analysis).

We thereafter introduced TV viewing and PA in the same model to examine their independent associations with individual and clustered metabolic-risk factors after further adjustment for adiposity (where adiposity was not the outcome of interest) (Table 3). In this model, there was a positive association between TV viewing and adiposity (*p* = 0.021). However, after adjustment for PA, gender, age group, study location, sexual maturity, smoking status, birth weight, and parental SES, the association of TV viewing with clustered metabolic risk was no longer significant (standardized β = 0.026, *p* = 0.053) (Figure 2).

**Table 3:**
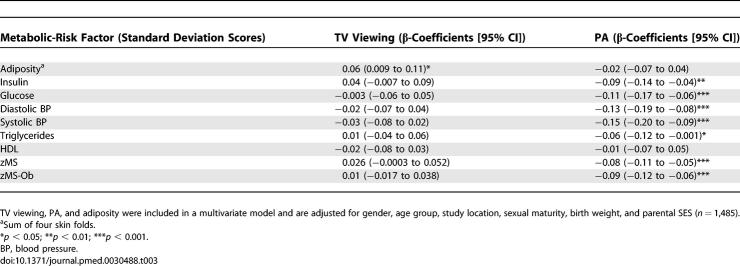
Independent Associations of TV Viewing and of Objectively Measured PA, with Individual and Clustered Metabolic-Risk Factors with zMS and with zMS-Ob

**Figure 3:**
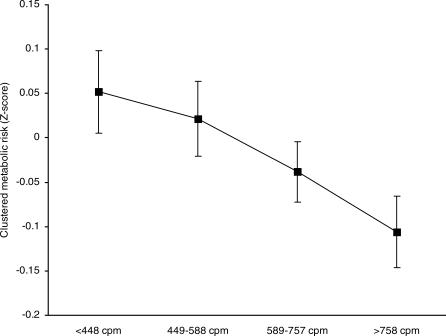
The Clustered Metabolic-Risk Score (Mean 95% CI) Stratified by Quartiles of PA (*n* = 1,485) Data on PA are adjusted for TV viewing, gender, study location, age group, sexual maturity, smoking status, birth weight, and parental SES (*p* < 0.0001 in continuous analysis).

The associations between PA with fasting insulin (*p* < 0.008), glucose (*p* < 0.001), triglycerides (*p* < 0.007), systolic and diastolic blood pressure (*p* < 0.001) were slightly attenuated but remained statistically significant after further adjustment for TV viewing and adiposity (Table 3). Furthermore, PA was significantly and inversely associated with the metabolic-risk score after adjustment for TV viewing, adiposity, and the same confounders as above (standardized β = −0.08, *p* < 0.0001) (Figure 3; Table 2).

We then examined the independent associations between TV viewing, PA, and the metabolic-risk score by excluding the adiposity component from the summary score and adjusting for adiposity as a confounder to examine whether the associations observed were independent of adiposity. PA (β = −0.09, *p* < 0.0001), but not TV viewing (β = 0.01, *p* = 0.46) was significantly associated with the metabolic-risk score, independently of adiposity. No significant interactions were observed between main exposures in any of our models.

We thereafter tested whether there was a difference in clustered metabolic-risk score between excluded and non-excluded children, but there was no difference (*p* = 0.871). Similarly, there was no difference in the mean sum of skin folds between excluded and non-excluded children (*p* = 0.98). It is therefore unlikely that the exclusion of children with missing activity data will bias our results. Finally, we reanalysed all our data, adjusting only for gender, age group, and study location (*n* = 1,702), and the results were unchanged.

Since the association between TV viewing and the clustered metabolic-risk score appeared to be mediated by adiposity, we examined whether self-reported frequency of eating meals while viewing TV influenced the association between TV viewing and adiposity in post hoc analysis. TV viewing was associated with frequency of eating meals while viewing TV. Further adjustment for eating while watching TV attenuated the association between TV viewing and adiposity (β = 0.043, *p* = 0.055). Furthermore, eating meals while viewing TV was significantly and positively associated with adiposity after adjustment for gender, age group, study location, and PA (*p* = 0.029).

## Discussion

We have shown that the association of TV viewing with clustered metabolic-risk factors appears to be mediated by adiposity. In contrast, PA was significantly and inversely associated with most individual metabolic-risk factors and with clustered metabolic risk, independently of TV viewing, adiposity, and other confounders. Our results appeared not to be modified by gender or age group and suggest the existence of separate associations between both TV viewing and PA, and individual and clustered metabolic-risk factors.

There are several limitations that should be considered when interpreting the findings from this study. It is difficult to infer a causal relationship, and it is impossible to determine its direction from cross-sectional data. Furthermore, only randomization within a trial can deal with issues of unmeasured confounding. Although we controlled for several potential confounders such as gender, age group, study location, sexual maturity, smoking status, birth weight, and parental SES, we cannot be certain that other unmeasured confounders, such as total energy intake, genetic variation, and other socio-cultural factors could not explain our observations.

TV viewing was measured by self-reporting, and the respondents were asked about TV viewing before and after school on an average weekday. It is difficult to assess the validity of these self-reports. However, the observed associations between TV viewing and individual risk (i.e., adiposity) and clustered metabolic risk are unlikely to be due to measurement error. Firstly, weekday TV viewing is suggested to be a reasonable indicator of overall TV viewing in children [9]. Secondly, measurement error could only explain the observed associations if there was a systematic bias, such as if those who were overweight over-reported their time spent viewing TV, and if those who were of normal weight underreported their time spent viewing TV. It is, however, plausible that overweight children underreport the amount of time spent viewing TV, similar to that which has been observed for food intake [23,24]. If this is the case, it may then underestimate the true association between TV viewing and individual and clustered metabolic risk. Finally, our exposures are likely to be measured with different degrees of error, which will differently attenuate the true associations. Unfortunately, repeated measurements of our exposures are not available, which precludes the possibility of correcting our analyses for measurement error.

The strengths of our study include our large population-based sample, our validated method for measuring PA by accelerometry, the collection of fasting blood samples in a large group of children, the use of skin-fold measurements for assessing adiposity, and the use of a computer-based questionnaire for assessing self-reported variables. Finally, few if any previous studies have examined the joint association of objectively measured PA and TV viewing with regard to metabolic-risk factors in children, taking into account the potential confounding effect of obesity as well as other known confounding factors.

Other researchers have found TV viewing to be associated with obesity and some metabolic-risk factors [4–9,25], and suggested that TV viewing maybe an indicator of sedentary behaviour. However, we did not observe any association between TV viewing and PA, and the association between TV viewing and adiposity was independent of PA. This suggests that TV viewing does not displace PA and that other factors, such as dietary behaviour and quality while viewing TV, may influence energy balance and thereby body weight. It has also been suggested that TV viewing has a lowering effect on the metabolic rate in children [26], but the data are not conclusive [27,28]. In post hoc analyses, we observed that the self-reported frequency of eating meals while viewing TV attenuated the association between TV viewing and adiposity. Furthermore, self-reported eating frequency while viewing TV was associated with adiposity independently of gender, age group, study location, and PA. Decreasing targeted sedentary behaviour, including TV viewing, significantly decreases energy intake in youth, whereas increasing sedentary behaviour did not affect energy intake [29]. Furthermore, eating between meals is consistently associated with TV viewing [30] and snacking, while watching TV is associated with increased total energy intake and energy intake from fat in particular [31]. Taken together, this suggests that TV viewing is associated with increased energy intake, which may affect energy balance and subsequent weight gain in children. However, it is also possible that TV viewing is increased as a result of being more overweight.

PA explains a low amount of the variance in obesity in youth [16,32], and PA is weakly associated with weight gain [33]. Reducing TV viewing is likely to prevent weight gain either directly or indirectly. The American Academy of Pediatrics suggests that TV viewing should be limited to 1–2 h d^−1^ in children [34], although data suggest that less than 1 h is even better [9]. Our data suggest that TV viewing, but not PA, is associated with adiposity, whereas PA is associated with other metabolic-risk factors. Preventive strategies may therefore need to target these two behaviours separately.

We have previously observed a significant inverse association between objectively measured PA and clustered metabolic risk in a subgroup of our study participants [19]. Our current findings extend these observations to also include older children and children from different socio-cultural and geographical locations. The observed associations between PA and metabolic risk were strong and independent of TV viewing, adiposity, and other confounding factors. It is biologically plausible that PA improves the metabolic-risk profile without influencing adiposity. Firstly, PA improves insulin action and glucose transport [35]. Secondly, PA increases blood flow and oxygen supply through increased capillarization and vasodilatation by nitric oxide, which improves fat metabolism [36,37]. Thirdly, PA may affect sympathetic tone and thus blood pressure may decrease through a more efficient recruitment of the motor units in the muscle [38].

The individual risk factors assessed in this study are established risk factors for CVD in adult life. Therefore, identifying the associations of sedentary and PA behaviour with these risk factors in children may be essential for the primary and secondary prevention of all diseases that result from sedentary behaviour. In the present study, moving from one quartile of PA to the next (an increase of approximately 140 cpm d^−1^) equates to improvements in metabolic risk of about 0.053 standard deviation (Figure 3). Based on doubly-labelled water data [17,39], this amount of activity equates to energy expenditure through PA of about 30 kJ kg^−1^ d^−1^. For the average child in our study, this is approximately 1.5 MJ d^−1^, or about 50–60 min d^−1^ of moderate-intensity activity. This amount of activity can be accumulated through various activities and does not necessarily include structured exercise. A change of 0.053 standard deviation corresponds, for example, to a change in blood pressure of less than 0.1 mm Hg, a change in insulin of about 0.04 mmol l^−1^, or a change in triglycerides of about 0.003 mmol l^−1^. Some could regard this as clinically insignificant. However, this dose-response relationship was observed in a population of healthy children and, as metabolic-risk factors track over time, they may result in substantial reductions of the occurrence of disease later in life [3].

In conclusion, in relation to metabolic risk in children, TV viewing and PA should be considered as separate entities as they are differentially associated with individual and clustered metabolic-risk factors. Our data suggest that the association between TV viewing and clustered metabolic risk is mediated by adiposity, whereas PA is associated with individual and clustered risk independently of TV viewing and adiposity. These observations may provide separate opportunities for prevention against obesity and metabolic risk in children.

## Supporting Information

Alternative Language Abstract S1 Translation of the Abstract into Swedish by Ulf Ekelund

## Abbreviations

ANOVA: analysis of variance
BMI: body-mass index
CI: confidence interval
cpm: counts per minute
CVD: cardiovascular disease
EYHS: European Youth Heart Study
HDL: high-density lipoprotein
PA: physical activity
SES: socio-economic status
WHO: World Health Organization
zMS: standardized metabolic-risk score
zMS-Ob: standardized metabolic-risk score excluding the adiposity component
