# Supplementary material for: TV Viewing and Physical Activity Are Independently Associated with Metabolic Risk in Children: The European Youth Heart Study
Source: PLoS Med. 2006 Dec 12;3(12):e488. doi: 10.1371/journal.pmed.0030488 (PMC1705825; doi:10.1371/journal.pmed.0030488)
Supplement: Supplementary file 1 [file pmed.0030488.sd001.doc]

**PloS Medicine (06-PLME-RA-0438R1)**

**Swedish abstract**

**Bakgrund**: Tidigare studier har föreslagit ett samband mellan TV tid och metabola riskfaktorer bland barn och unga. Det är emellertid oklart om detta samband är oberoende av fysisk aktivitet och fettmassa.

**Metod och resultat**: I en populations-baserad tvärsnittstudie (European Youth Heart Study) omfattande 9-10 och 15-16 år gamla pojkar (n = 911) och flickor (n = 1010) från tre geografiskt skilda regioner (Madeira, Estland och Danmark) undersökte vi sambandet mellan TV tid, objektivt mätt fysisk aktivitet (med accelerometer) och metabola riskfaktorer (blodtryck, glukos, insulin, triglycerider, HDL kolesterol, samt mängden kroppsfett). Metabol risk uttrycktes som en kontinuerlig, normalfördelad variabel genom att beräkna medelvärdet av de standardiserade värdena för de sex ingående delkomponenterna.

Det förelåg inget samband mellan TV tid och fysisk aktivitet. TV tid var relaterat till metabol risk (P = 0.053) efter att hänsyn tagits till fysisk aktivitet, kön, ålder, region, pubertersstatus, rökning, födelsevikt och föräldrarnas socio-ekonomiska status. Detta samband förklarades av ett positivt samband mellan TV tid och och fettmassa (P = 0.021). Vidare analyser antydde att ätbeteendet vid TV tittande förklarade sambandet mellan TV tid och fettmassa. Fysisk aktivitet var signifikant och oberoende relaterat till systoliskt och diastoliskt blodtryck, glukos, insulin (P < 0.01), och triglycerider (P = 0.02). Fysisk aktivitet var också signifikant och oberoende relaterat till metabol risk (P < 0.0001).

**Slutsats:** Det föreligger ett komplext samband mellan TV tid, fysisk aktivitet och metabola riskfaktorer. TV tid och fysisk aktivitet är inte relaterat till varandra och olika relaterat till metabol risk. Sambandet mellan TV tid och metabol risk medierades av fettmassa som i sin tur möjligen förklaras av ätbeteendet då unga tittar på TV. Fysisk aktivitet är starkt och oberoende relaterat till metabol risk men inte till fettmassa. Våra resultat tyder på att förebyggande åtgärder bland barn och unga måste inriktas mot ätbeteendet då de tittar på TV samt mot att öka graden av fysisk aktivitet för att ha optimal effekt.
